# Versatile optoelectronic memristor based on wide-bandgap Ga_2_O_3_ for artificial synapses and neuromorphic computing

**DOI:** 10.1038/s41377-025-01773-6

**Published:** 2025-04-15

**Authors:** Dongsheng Cui, Mengjiao Pei, Zhenhua Lin, Hong Zhang, Mengyang Kang, Yifei Wang, Xiangxiang Gao, Jie Su, Jinshui Miao, Yun Li, Jincheng Zhang, Yue Hao, Jingjing Chang

**Affiliations:** 1 Advanced Interdisciplinary Research Center for Flexible Electronics, Academy of Advanced Interdisciplinary Research, Xidian University, 710071 Xi’an, China; 2 State Key Laboratory of Wide-Bandgap Semiconductor Devices and Integrated Technology, School of Microelectronics, Xidian University, 710071 Xi’an, China; 3 National Laboratory of Solid-State Microstructures, School of Electronic Science and Engineering, Collaborative Innovation Center of Advanced Microstructures, Nanjing University, 210093 Nanjing, China; 4 State Key Laboratory of Infrared Physics, Shanghai Institute of Technical Physics, Chinese Academy of Sciences, Shanghai, 200083 China

**Keywords:** Electronics, photonics and device physics, Optical physics

## Abstract

Optoelectronic memristors possess capabilities of data storage and mimicking human visual perception. They hold great promise in neuromorphic visual systems (NVs). This study introduces the amorphous wide-bandgap Ga_2_O_3_ photoelectric synaptic memristor, which achieves 3-bit data storage through the adjustment of current compliance (*I*_cc_) and the utilization of variable ultraviolet (UV-254 nm) light intensities. The “AND” and “OR” logic gates in memristor-aided logic (MAGIC) are implemented by utilizing voltage polarity and UV light as input signals. The device also exhibits highly stable synaptic characteristics such as paired-pulse facilitation (PPF), spike-intensity dependent plasticity (SIDP), spike-number dependent plasticity (SNDP), spike-time dependent plasticity (STDP), spike-frequency dependent plasticity (SFDP) and the learning experience behavior. Finally, when integrated into an artificial neural network (ANN), the Ag/Ga_2_O_3_/Pt memristive device mimicked optical pulse potentiation and electrical pulse depression with high pattern accuracy (90.7%). The single memristive cells with multifunctional features are promising candidates for optoelectronic memory storage, neuromorphic computing, and artificial visual perception applications.

## Abstract (web summary)

**Multi-functional integration!** The memristor integrates four functions of multi-level storage, logic gates, UV sensing, and neuromorphic computing in one device.

## Graphical abstract

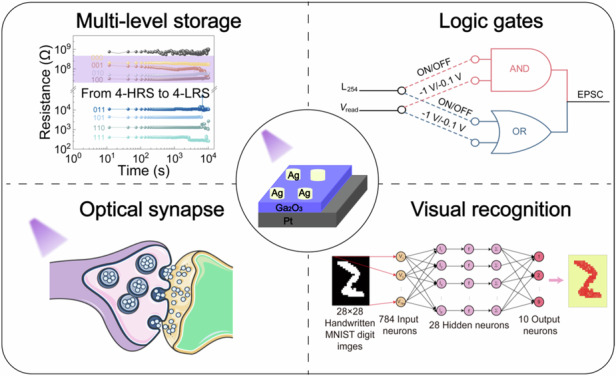

## Introduction

The information technology industry has increased its demand for chip arithmetic power and energy efficiency due to the need to process large amounts of data quickly and effectively. The separation of data storage and computing components in the traditional von Neumann architecture gives rise to challenges such as “storage walls”^1^, leading to substantial extra energy consumption and transmission delays. Consequently, scientists are creating innovative devices that mimic the human brain, allowing storage and processing in a single system. Neuromorphic computing, a type of computing, continues to drive the development of future computing systems^2^. Resistive switching-based memristors, providing the benefits of low power consumption, multifaceted switching, fast switching speeds, and tiny cell sizes, are suitable for conventional complementary metal-oxide semiconductor (CMOS) technologies^3–5^. Meanwhile, the storage and neuromorphic computation capabilities of the resistive switching-based memristors, make it the preferred option for non-volatile and neuromorphic computing applications^6,7^. Up to now, neuromorphic computation has been implemented with a variety of materials, including organic semiconductors^8^, perovskite^9^, 2D materials^10^, and oxide semiconductors^11^. These materials provide a broad optical response spanning from the ultraviolet (UV) to near-infrared (NIR) region, restricting their applications in solar-blind detection^12^. Meanwhile, the wide bandgap energy and excellent optical and electrical properties of Ga_2_O_3_ make it a suitable oxide semiconductor for resistive switching. Additionally, Ga_2_O_3_ has excellent radiation hardness, thermal and chemical stability, as well as efficient adsorption in solar blind regions, making it suitable for applications in extreme environments such as aerospace and space exploration^13^. And Ga_2_O_3_ thin film phototransistors and photodetectors have been used to achieve neuromorphic visual systems (NVs). So far, there are few studies of resistive-switching-based memristors based on wide-bandgap Ga_2_O_3_. Previously, Long et al. described an optoelectronic memristor computing system that uses an amorphous GaO_x_-based photo-synapse to recognize latent fingerprints with excellent efficiency^14^. Xu et al. investigated Sn-doped Ga_2_O_3_ optoelectronic devices with high responsivity and extended response decay time, which might mimic photonic synaptic behaviors and picture pre-processing capabilities^12^. Based on the photodetector structure, these two works illustrate the photo-detector and photo-synapse functions. Qian et al. reported the 2D Ga_2_O_3_-based memristors that exhibit forming-free and bipolar switching capabilities. The application of electrical pulses revealed essential biological synapse functions, including PPF, LTP, and LTD^15^. Qian realized the functions of data storage and electrical synapses based on the structure of the memristor. According to the above research status, it can be seen that combining data storage and photoreceptors in a single device is challenging. Thus, it is highly desirable to search for devices that can integrate data storage, optical sensing, and biological synapses.

In this study, we fabricated wide-bandgap Ga_2_O_3_ thin film resistive random-access memories (RRAMs) with Ag/Ga_2_O_3_/Pt device configuration. This system integrates UV light sensors, data storage, logic gates, and neuromorphic computing in one single device. We adjusted the *I*_cc_ to achieve four low resistance states (LRS). Applying four distinct intensities of UV light (254 nm) with an *I*_cc_ of 1 mA allows for four different high resistance states (HRS). The resistance values of LRS remain fairly stable. Based on the above two methods, we have identified eight distinct resistance states that enable a device to achieve multilevel resistive switching (MRS) ability. Interestingly, the device demonstrates consistent volatility when the *I*_cc_ is adjusted to 1 µA. Subsequently, due to the bipolar nature of this memristor, the device exhibits reduced conductance when subjected to a negative bias voltage as opposed to a positive bias voltage. Hence, we consider the 254 nm light source and the bias voltage to be the two inputs of the device, with the current as the output. A positive bias voltage is used to implement the “OR” logic gate. An “AND” logic gate is implemented when a negative bias voltage is applied. Furthermore, the device was irradiated with a sequence of UV light pulses to achieve synaptic functions such as PPF, SIDP, SNDP, STDP, and SFDP. The advanced neuromorphic characteristics of learning-forgetting-relearning were successfully achieved. After obtaining conductance from 50 sets of light pulse potentiation and 50 sets of electrical pulse depression, conductance values are used as weights, and the modified National Institute of Standards and Technology (MNIST) dataset is used as the training input for the ANN. As a result, a maximum image recognition rate of 90.7% is attained.

## Results

### Multilevel resistive switching ability and volatile property

The microstructures and compositions of the Ga_2_O_3_ thin film were analyzed using X-ray Diffraction (XRD) and X-ray Photoelectron Spectroscopy (XPS) techniques. The XRD measurement result is depicted in Fig. S1a (Supplementary information). The XRD pattern in the 2θ range of 10°–50° reveals the absence of distinct Ga_2_O_3_ peaks. Instead, all observed peaks correspond to the Pt substrate, suggesting that the resulting Ga_2_O_3_ thin film is amorphous^16,17^. Fig. S1b displays the O 1 s core level spectrum of the Ga_2_O_3_ thin film. The peak fitting reveals two peaks with binding energies of 530.7 eV and 531.3 eV, respectively. The peak with a binding energy of 530.7 eV (66.36%) is attributed to lattice oxygen in the previous study^18–20^, whereas the other peak is attributed to an oxygen vacancy (33.64%). The wide-bandgap Ga_2_O_3_-based photoelectronic memristor with abundant oxygen vacancies exhibits a significant persistent photoconductivity (PPC) effect, which could be utilized and engineered for optoelectronic synapse function simulations^14,21^.

**Fig. 1:**
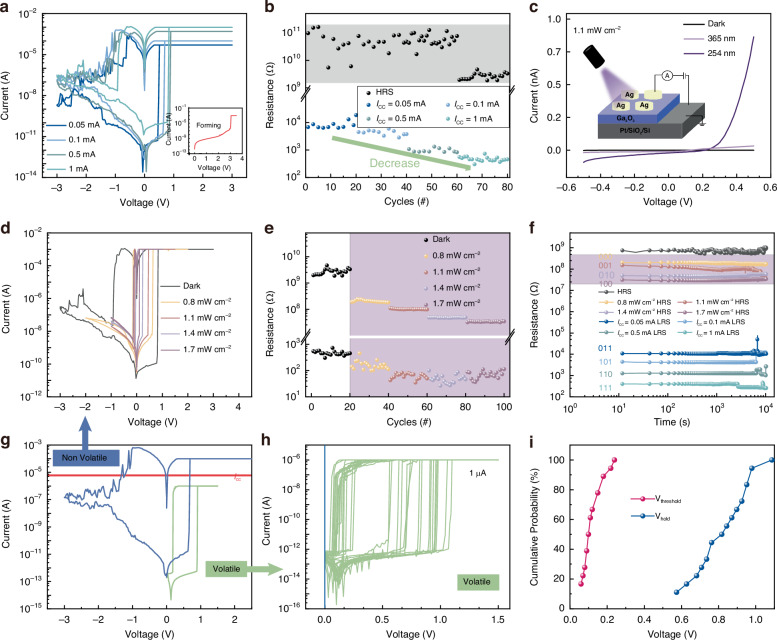
**Device characteristics of memristors. a** Typical *I*-*V* curves of the Ag/Ga_2_O_3_/Pt device under different *I*_cc_. **b** Resistance distribution of 80 cycles in HRS and LRS under different *I*_cc_. **c** The *I*-*V* curves under the dark, 254 nm light, and 365 nm light. **d** The typical *I*-*V* curves of the Ag/Ga_2_O_3_/Pt device under the different UV (254 nm) intensities with *I*_cc_ = 1 mA. **e** The resistance distribution of 20 cycles in HRS and LRS under different UV (254 nm) intensities. **f** The retention characteristics of eight different resistance states. **g** Coexistence of non-volatile and volatile during different *I*_cc_. **h** *I*-*V* curves of 20 scanning cycles with *I*_cc_ = 1×10^-6 ^A. **i** Cumulative probability statistics of *V*_th_ and *V*_hold_

Figure 1a shows the *I* − *V* characteristics of the memristor at various *I*_cc_ (1 mA, 0.5 mA, 0.1 mA, and 0.05 mA). To start the resistance-switching (RS) process, a + 3 V electroforming voltage was first given to the device (inset of Fig. 1a). To investigate the Ag/Ga_2_O_3_/Pt device’s RS reproducibility and switching voltage distribution, 80 consecutive *I*-*V* scans were performed (Fig. S2a–d, Supplementary Information). Additionally, the fabricated device demonstrated *I*_cc_-dependent RS, which is advantageous for multilevel LRS-memory cells (Fig. 1a). Figure 1b illustrates the resistance distribution of 20 cycles in HRS and LRS with *I*_cc_ ranging from 0.05 mA to 1 mA. The resistance of the LRS decreased as *I*_cc_ increased. The HRS resistance fluctuated with the range from 1.9 GΩ to 208 GΩ under different *I*_cc_, resulting in four low-resistance states and one high-resistance state. As *I*_cc_ increases, more conductive filaments are generated. As a result, when *I*_cc_ is 1 mA, the resistance in HRS drops proportionally. Fig. S3 shows the retention characteristic under various *I*_cc_, demonstrating that the four low-resistance states have good stability for more than 1000 s under various *I*_cc_. Meanwhile, the endurance and device-to-device performance are shown in Fig. S4a and Fig. S4b (Supplementary Information). The resistive switching mechanism is analyzed under different *I*_cc_ values, as illustrated in Fig. S5a–d (Supplementary Information). The device exhibits a conductive filaments mechanism in the low-resistance state (LRS) and a Schottky emission mechanism in the high-resistance state (HRS). Finally, based on this mechanism, a physical model of the resistive switching is proposed, as shown in Fig. S6a–c (Supplementary Information). The power consumption for multilevel storage is analyzed in Table S1 (Supplementary Information).

Due to the high responsivity of Ga_2_O_3_ films to deep UV light, the photo-response properties of the Ga_2_O_3_ films are studied. Fig. S7 depicts the absorbance of wide-bandgap Ga_2_O_3_ film at various light wavelengths, indicating that the fabricated wide-bandgap Ga_2_O_3_ films are highly responsive to deep UV light between 200 nm and 300 nm. The inset in Fig. 1c shows the device structure. The *I*-*V* curves of the Ag/Ga_2_O_3_/Pt device were recorded under dark, 254 nm and 365 nm UV (1.1 mW cm^–2^), respectively, with an applied bias from −0.5 V to 0.5 V (Fig. 1c). It can be seen that the response of the memristor to UV light with a wavelength of 254 nm is superior. When the light intensity increased from 0.5 mW cm^–2^ to 2.0 mW cm^–2^, the illuminated currents (365 nm and 254 nm) increased by one and three orders of magnitude, respectively, in Fig. S8a and Fig. S8b (Supplementary Information).

The *I*-*V* curves of the memristor were then tested under various light intensities (254 nm) and *I*_cc_ value of 1 mA, as seen in Fig. 1d. The fabricated device exhibited light intensity-dependent HRS (Fig. 1d), which is ideal for multilevel HRS memory cells. We increased the light intensity from 0.8 mW cm^–2^ to 1.7 mW cm^–2^ to illustrate the device’s MRS capacity. As the light intensity grew, the current in the HRS increased, resulting in four high-resistance state levels and one low-resistance state level. Eighty successive *I*-*V* scans were performed to examine the switching voltage distribution and RS reproducibility under UV light at 254 nm. The results are displayed in Fig. S9a-d (Supplementary Information). The resistance distribution of 20 cycles in HRS and LRS under UV intensities ranging from 0.8 mW cm^-2^ to 1.7 mW cm^-2^ is shown in Fig. 1e. The resistances of the LRS varied within the range from 27 Ω to 455 Ω under different light intensities. The HRS resistances decreased with increased UV intensity, leading to four high-resistance states and one low-resistance state. Fig. S10 shows the retention characteristics under different UV intensities. This demonstrates that the four high-resistance states obtained at various UV intensities can remain stable for over 1000 s. Combining the four low-resistance states obtained under different *I*_cc_ and four high-resistance states obtained under different UV intensities, the optoelectronic memristor has eight different resistance states as shown in Fig. 1f. As a result, the constructed Ag/Ga_2_O_3_/Pt device can store three bits per memory cell, tripling the memory system’s storage density^22^.

In order to obtain more states of the resistance in LRS, we reduced *I*_cc_ to 1 µA. Interestingly, the memristor exhibits a volatile characteristic with *I*_cc_ = 1 µA^23^, as shown in Fig. 1g. The current abruptly climbs to the *I*_cc_ value at approximately 0.91 V, indicating that the device has transitioned from the HRS to the LRS, and then begins to fall at approximately 0.18 V. The device has volatile threshold RS features, and the random threshold switch (TS) behavior makes it suitable for replicating neurons’ leak integration-and-fire function^24–26^. In addition, the volatile threshold RS characteristics of the Ag/Ga_2_O_3_/Pt device were investigated by 20 scanning *I*-*V* cycles, and the distributions of *V*_th_ and *V*_hold_ are shown in Fig. 1h, i, respectively. Fig. S11 (Supplementary Information) shows the *I*-*V* curves illustrating the volatility characteristics of 20 devices, demonstrating the robustness of this characteristic. The devices exhibit stable volatility at *I*_cc_ = 1 µA.

### Logic gates

**Fig. 2:**
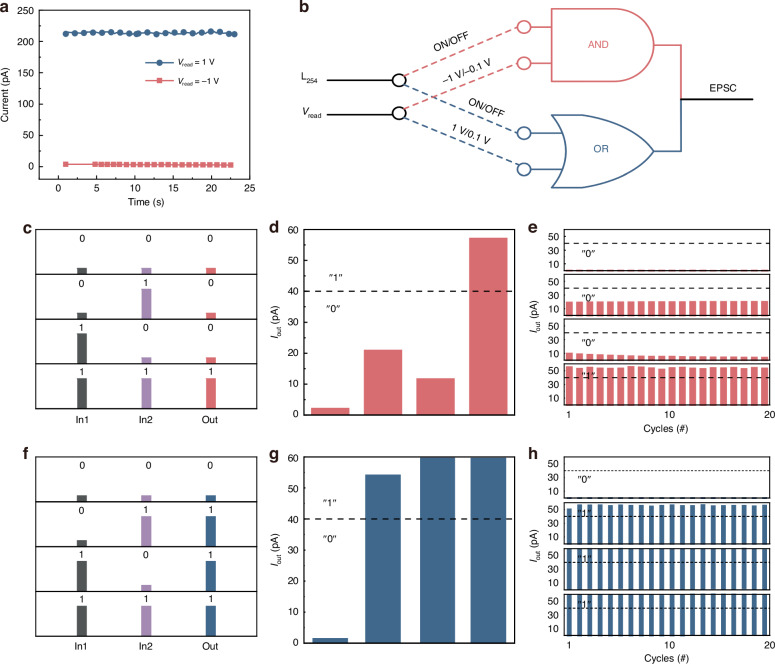
**Architecture and performance of Logic gates. a** The different currents under the read voltage of –1 V and 1 V. **b** Schematic of “AND” logic gate and “OR” logic gate. (In1 is the read voltage, and the In2 is the UV light of 254 nm.) **c** The “AND” logic gate truth table, In1 is the read voltage of –1V (“1”) or –0.1 V (“0”), and In2 is the UV light of ON/OFF. **d** The typical result, and (**e**) the experimental results of the “AND” logic gate in 20 cycles. **f** The “OR” logic gate truth table, In1 is the read voltage of 1 V (“1”) or 0.1 V (“0”), and In2 is the UV light of ON/OFF. **g** The typical result, and (**h**) the experimental results of the “OR” logic gate in 20 cycles

Memristor-assisted logic is a significant subset of the logic family that distinguishes itself by the segregation of inputs and outputs and the lack of additional peripheral circuitry^27,28^. The device exhibits lower conductance at negative bias voltage than at positive bias voltage as illustrated in Fig. 2a. “AND” and “OR” logic gates can be implemented at these two distinct bias voltages, respectively, as shown in Fig. 2b. The memristor-based logic gate utilizes both electrical and optical signals as input. In the “AND” gate, Input1 in Fig. 2c is assigned a negative value. Specifically, -1 V corresponds to the logical value “1”, whereas –0.1 V corresponds to the logical value “0”. Input2 is a 254 nm UV light source, with light on representing “1” and light off representing “0”. Figure 2d displays the output of a standard “AND” gate. The output current is classified as “1” if it exceeds 40 pA and “0” if it falls below 40 pA. Figure 2e depicts the output of the “AND” gate over 20 cycles, which is consistent well with the output shown in Fig. 2d. This suggests that the logic function of the device is relatively stable.

Figure 2f depicts the “OR” gate. The input voltage for a positive logic operation is 1 V, representing the logic operation of “1”. A voltage of 0.1 V represents the logic operation of “0”. Input 2 is a 254 nm UV light source, with light on representing “1” and light off representing “0”. Figure 2g displays a standard output of an “OR” gate. The output current is classified as “1” if it exceeds 40 pA and “0” if it falls below 40 pA. Figure 2h displays the output of the “OR” gate over 20 cycles, exhibiting a high level of consistency with the output seen in Fig. 2g. The power consumption of logic gate functions is analyzed in Table S2 (Supplementary Information). The total power consumption required for implementing “AND” and “OR” logic gates is 61.36 pW and 544.89 pW, respectively.

### Optical synaptic plasticity

**Fig. 3:**
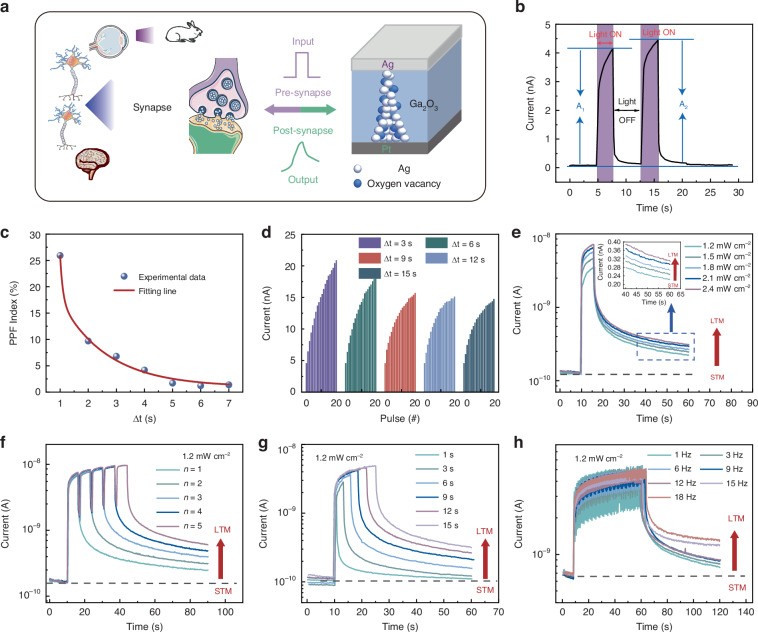
**Optoelectronic synapse based on the optical response of Ga**_**2**_**O**_**3**_**. a** Schematic depiction of the human visual system, including image sensing, memory, and processing, as well as an Ag/Ga_2_O_3_/Pt synaptic device. **b** PSC responses are activated by two consecutive UV light pulses. **c** PPF index fitting results plotted by pulse intervals Δt. **d** The variations of PSC in 20 cycles of different Δt (3 s–15 s). The transformation from STM to LTM in PSC. **e** intensity, (**f**) number of light pulses, (**g**) duration, (**h**) frequency. (UV light: 254 nm)

The schematic of the bio-synapse, a fundamental component of the biological vision system, is depicted in Fig. 3a. The bio-synapse acts as a connecting channel between two neurons and facilitates the information transfer from pre-neuron to post-neuron via electrical or electrochemical impulses^21,29^. In biological nervous systems, the capacity of synapses to increase in response to two consecutive pulses is known as PPF, a basic characteristic of synaptic short-term plasticity (STP). Such enhancement is a function of the interval between the two pulses, increasing as the interval decreases. PPF performance is crucial for encoding temporal data in both visual and auditory sources. It also serves as a foundation for cerebral functions such as information processing, pattern recognition, sound source localization, and memory learning^30–32^. The photo-responsive currents generated by two successive light pulses (5 s time interval) are depicted in Fig. 3b. The amplitudes of post-synaptic-current (PSC) caused by the first and second light pulses are indicated as A_1_ and A_2_, respectively, and (A_2_-A_1_)/A_1_ was employed to calculate the PPF index^33^. A larger PSC was stimulated by the second light pulse than the first pulse. This is because the photonic current generated by the first light stimulus has not fully decayed to its original state when the second one was applied. Figure 3c demonstrates that the PPF index is enhanced from 2% to 27% gradually, as Δt goes down from 7 s to 1 s, relating to Δt closely. Meanwhile, the double exponential decay function was employed to further investigate the relationship between the experimentally obtained PPF index and Δt. The data are well-fitted and the fitting equation is shown below:

1 $${\rm{PPF}}={A}_{0}+{A}_{1}\exp \left(-\frac{\Delta t}{{\tau }_{1}}\right)+{A}_{2}\exp \left(-\frac{\Delta t}{{\tau }_{2}}\right)$$

where τ_1_ and τ_2_ represent the rapid and slow PPF decay periods, respectively, A_1_ and A_2_ indicate the fast and slow facilitation magnitudes, respectively^34,35^. Here, τ_1_ and τ_2_ correspond to the synaptic time scale of 0.1 s and 1.7 s, respectively^21,36,37^. Spike trains can extend such PPF function into spike rate dependent plasticity (SRDP) function (Fig. 3d). One spike train consists of 20 successive UV spikes (1.8 mW cm^-2^, spike width 3 s). The spike rate was varied when changing the spike interval from 3 s to 15 s. Similar to the biological synapses’ SRDP function, raising the spike rate produces much higher Excitatory Postsynaptic Currents (EPSCs).

We realized short-term memory (STM), long-term memory (LTM), and STM to LTM transition processes in wide-bandgap Ga_2_O_3_-based artificial optoelectronic synapses by varying the intensity, number, duration, and frequency of light pulses, as shown in Fig. 3e–h. Figure 3e illustrates the relationship between PSC and the intensity of a light pulse, which is known as SIDP. By increasing the strength of the light pulse from 1.2 mW cm^–2^ to 2.4 mW cm^–2^ (with a pulse duration of 6 s) and stopping the light pulse for 30 s, the PSC amplified from 0.22 nA to 0.31 nA, representing an increase of 183.3% and 246.2% compared to the original state respectively. SNDP, as demonstrated by the fluctuation in PSC with the number of light pulses, is depicted in Fig. 3f. The PSC of the Ga_2_O_3_-based optoelectronic memristor exhibits a positive correlation with the number of light pulses. As light pulses increase from one to five, the PSC rises from 0.25 nA to 0.6 nA. A link known as STDP is depicted in Fig. 3g between the fluctuation of PSC and the width of the light pulse. Furthermore, we observed the transition from STM to LTM by extending the light pulse duration. It has been discovered that the PSC increases from 0.11 nA to 0.32 nA as the light irradiation period increases, while the decay rate decreases. Figure 3h illustrates the correlation between the PSC and the frequency of light pulses, referred to as SFDP. Seven distinct pulse frequencies were utilized, each with a 50-second light pulse duration and 1.2 mW cm^-2^ light intensity. It is discovered that the PSC grows in tandem with the frequency of the light pulse. The PSC changed little at pulse frequencies below 12 Hz, while increased significantly when pulse frequencies exceeded 15 Hz. Energy consumption is critical for artificial synapses. Table S3 (Supplementary Information) provides detailed information about the energy consumption associated with the four synaptic events mentioned above. The average energy consumptions for four synaptic events, SIDP, SNDP, STDP, and SFDP, are 1.01$\times$10^–10 ^J µm^–2^, 7.20$\times$10^–11 ^J µm^–2^, 9.20$\times$10^–11 ^J µm^–2^, and 3.00$\times$10^–10 ^J µm^–2^, respectively.

These four activities appear to enhance PSC formation and memory retention. Hence, by employing four distinct light stimuli, artificial photoelectron-synapses based on wide-bandgap Ga_2_O_3_ effectively achieved the transition from STM to LTM, thereby confirming the replication of human visual memory function. The synaptic devices were also evaluated utilizing 365 nm UV light under the previous parameters. According to the results, the devices had only a simple photo-response under the 365 nm UV light (Fig. S12a–d, Supplementary Information).

### Learning experience

**Fig. 4:**
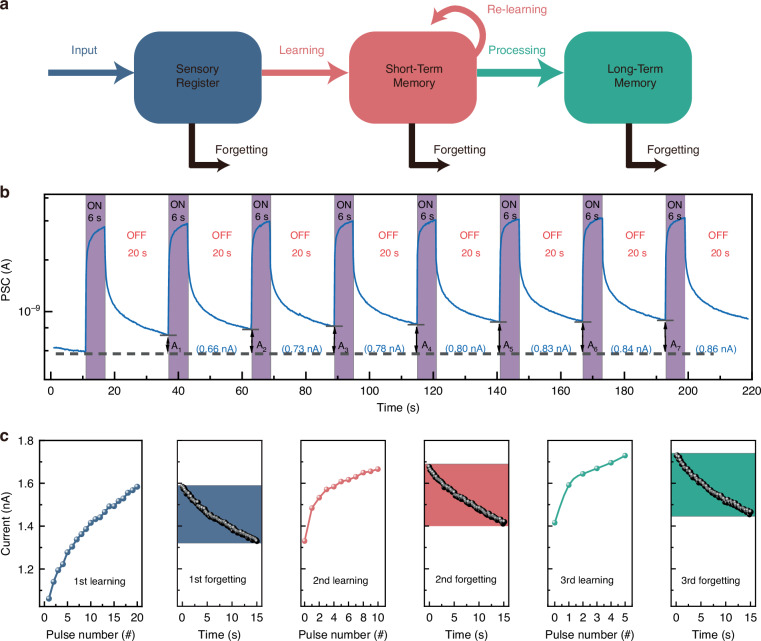
**Learning-forgetting-relearning behavior. a** Schematic diagram of the “learning-forgetting-relearning” behavior. **b** Learning-forgetting-relearning behavior with seven cycles. **c** The “learning experience” behavior was measured under UV light pulse (254 nm) stimulation. Light intensity: 1.8 mW cm^–2^, pulse width: 3 s, pulse interval: 3 s

“Learning-forgetting-relearning” behavior is a comprehensive memory storage model that includes both STM and LTM, which are the two primary types of memory in psychology (Fig. 4a)^38^. The hippocampus is responsible for storing STM. Memory is facilitated by temporary and subtle strengthening of synaptic connections, which can last for seconds or minutes before totally vanishing. Repeated reinforcement training allows STM information to be converted into LTM and then transferred to the cerebral cortex. This process entails a persistent strengthening of synaptic weights, which generally endures for hours, years, or even a lifetime. We established the optoelectronic memristor mimics the process of learning-forgetting by repeatedly activating and deactivating the UV light (254 nm), as depicted in Fig. 4b. In this case, turning on the light depicts the device’s learning and relearning activity while shutting it off represents forgetting. The PSC of the device exhibited a positive correlation with the number of light pulses, followed by a subsequent decay to an intermediate level after a specific duration. This observation implies a gradual loss of learned information over time. The PSC conductance of the device showed a small enhancement (A7 > A6 > A5 > A4 > A3 > A2 > A1) after repeated learning or relearning, indicating that early acquired knowledge significantly enhances memorization ability. The optoelectronic memristor successfully transitions from STM to LTM, as evidenced by the PSC conductance reaching its maximum level (A7) after seven consecutive learning and re-learning cycles. The simulation of superior synaptic function in synaptic plasticity, as reflected by the “learning-forgetting-relearning” process, became much easier due to the remarkable repeatability of the PSC response.

Furthermore, we demonstrate the learning-forgetting-relearning process of wide-bandgap Ga_2_O_3_-based photoelectric memristors by varying pulse numbers (20, 10, and 5), while maintaining a consistent forgetting period (15 s). The Ga_2_O_3_-based optoelectronic memristor was exposed to three sets of UV light pulses (254 nm), each with intensities of 1.8 mW cm^-2^, pulse widths of 3 s, and pulse intervals of 3 s. These three groups of light pulses have quantities of 20, 10, and 5, which correspond to three memory processes. The interval between each series of light pulses was 15 seconds, corresponding to the duration of memory decay for the three memory processes. Gradually, learning was successfully simulated by the synaptic device, as shown in Fig. 4c. Initially, 20 light pulses were applied to the device, which represented the “first learning” of the synapse. The 1st and 20th PSC amplitudes were measured to be 1.06 nA and 1.58 nA, respectively. The PSC increased as the number of pulses increased, suggesting an enhancement in the synaptic device’s learning effect (the synaptic weight). Within 15 seconds of the pulse being removed, the PSC diminished to 1.33 nA, indicating a weakening of synaptic weights. This phase corresponds to the forgetting process of the synaptic device.

The synaptic device’s “second learning” process was then demonstrated by applying 10 light pulses with the same settings. After the fifth pulse, the synaptic current is 1.58 nA, indicating that the synaptic weights have returned to the first learning process. The PSC is 1.67 nA after the tenth pulse, indicating that the synaptic device has progressed to a higher memory level. After forgetting for 15 s, the synaptic current fell to 1.42 nA. A higher current than the first learning-forgetting session suggests that the learning progress of the synaptic device has improved.

Furthermore, five light pulses under identical settings were used to symbolize the “third learning” process of the synaptic device. When the first pulse is administered, the PSC is 1.59 nA, suggesting that synaptic weights have returned to the first memory level. The PSC reached 1.67 nA after the third pulse, demonstrating that the synaptic weights had attained the second memory level. Following the fifth pulse, the PSC increased to 1.73 nA, signifying a further improvement in the memory capacity of the synaptic device. After 15 seconds of forgetting, the PSC reduced to 1.47 nA, indicating that the memory level was further strengthened.

As the 3rd learning process progressed, fewer light-pulse stimuli (20, 5, 1) were utilized to produce an equivalent PSC. The current declined significantly across the three 15 s forgetting cycles (from 1.33 nA to 1.47 nA). This behavior is similar to the phenomenon in which individuals tend to regain previously learned knowledge more quickly and this process of relearning can greatly improve the stability of memory^39,40^. By repeating this learning-forgetting-relearning process and rationally designing the period of each learning stage or number of pulses, synaptic weights can be stimulated to attain the learning objective, which is the desired memory level expressed as a certain current value. When the device is examined at a 365 nm UV light using the same test procedure, it is discovered that there is no learning memory behavior and it only responds at 365 nm (Fig. S13a, b, Supplementary Information).

### Visual recognition

**Fig. 5:**
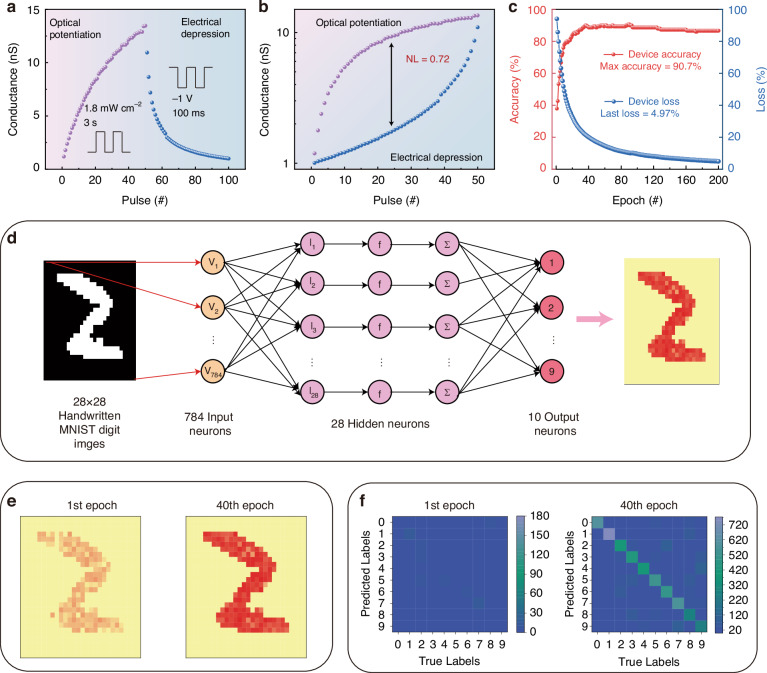
**Handwritten digit recognition with ANN. a** Optical potentiation and electrical depression behaviors. **b** The nonlinearity of weight update (NL) under the optical potentiation and electrical depression behaviors. **c** The recognition accuracy increases and the loss decreases with the training epochs. **d** The ANN network structure consists of input, hidden, and output layers. **e** The mapping images of the output number “2” after training. **f** Confusion matrix with the 1st training epoch and the 40th training epoch

In NVs (neuromorphic vision processing units), it is desirable to have high-level image processing capabilities with cognitive functionality to abstractly represent sensory input, which is especially useful in pattern recognition, image categorization, and localization^41,42^. Additionally, the pattern recognition capability of Ga_2_O_3_-based photoelectric memristors was also investigated. The weight update protocol linearity and symmetry have a significant impact on recognition accuracy in neuromorphic computing^43^. Before implementing the pattern recognition function, it is necessary to validate the optical pulse potentiation and electrical pulse depression capabilities of this memristor. The behavior of 50 optical pulse potentiation and 50 electrical pulse depression is shown in Fig. 5a, and a highly linear memristor is exhibited in Fig. 5b. The weight update nonlinearity (NL) evolution can be calculated by the equation as follows:

2 $${\rm{NL}}=\frac{\max \left|{{\rm{G}}}_{{\rm{P}}}^{i}-{{\rm{G}}}_{{\rm{D}}}^{i}\right|}{{{\rm{G}}}_{\max }-{{\rm{G}}}_{\min }}$$

Regarding the optical potentiation and electrical depression processes, the device conductance under i_th_ pulse stimulation is represented by ${{\rm{G}}}_{{\rm{P}}}^{i}$ and ${{\rm{G}}}_{{\rm{D}}}^{i}$ in Eq. (2), with the pulse number i ranging from 1 to 50. *G*_min_ represents the minimum conductance in the initial state, and *G*_max_ indicates the maximum conductance after 50 optical pulses. The nonlinearity of the Ga_2_O_3_-based photoelectric memristor is determined to be 0.72 as depicted in Fig. 5b, satisfying the requirement of the highly effective neuromorphic computing realization^12^.

Figure 5d shows the schematic of a three-layer ANN for a “2” input pattern recognition process. The neuromorphic computer system contains 784 input neurons associated with an MNIST image with dimensions of 28 × 28 pixels, 28 hidden neurons, and 10 output neurons. Each input neuron corresponds to a single pixel of the image. The 10 output neurons are associated with 10 distinct categories of numerical digits ranging from 0 to 9. The ANN was trained using 2850 patterns randomly chosen from the MNIST collection^44^. Following the training, recognition accuracy was evaluated using a distinct set of 150 patterns from the dataset. The simulations demonstrated the accuracy of the Ga_2_O_3_-based optoelectronic memristor across 200 epochs as depicted in Fig. 5c. The recognition accuracy increased significantly initially with a maximum accuracy of 90.7%. The loss of optical-electronic weight decreased over 20 epochs with the minimum loss reaching 4.97%. Energy consumption is critical in artificial neuromorphic systems. The energy consumptions of the optical potentiation and electrical depression in neuromorphic computation are 5.40$\times$10^–11 ^J µm^–2^ and 4.84$\times$10^–15 ^J µm^–2^, respectively, as shown in Table S4, S5 (Supplementary Information).

The mapping images of the output number “2” obtained after the training process are displayed in Fig. 5e. These images were generated using MATLAB software based on the recognition accuracy^45^. Using the number “2” as an example, we can see that the learned number was blurry and thinner during the first epoch. Following the training process, the training number acquired a more distinct shape, free from gaps, resulting in a recognition accuracy of 88.7% after 40 training epochs. The confusion matrix for the initial state and after training for the 1st and 40th epochs is shown in Fig. 5f. The simulation specifics of the ANN can be found in Table S6 (Supplementary Information). The matrix transferred from disorder to a standardized state, with each line representing a reduction in recognition errors and the gradual acquisition of knowledge by the optoelectronic synapse. The experimental outcome emphasizes the cognitive capacity of our optoelectronic synapse and its potential application for image recognition in neuromorphic computing. Furthermore, Table S7 (Supplementary Information) compares our proposed work with previously reported studies. The work presented herein focuses on the device’s superior capabilities in multilevel storage, AND/OR logic gate implementation, multi-pulse modulation of photo synaptic behavior, and neuromorphic computing applications. This design effectively integrates digital storage, logic operations, photo synapses, and neuromorphic computation within a single device. This innovative architecture provides new insights and opportunities for the application of two-terminal memristors in multifunctional neuromorphic computation.

## Discussion

The wide-bandgap Ga_2_O_3_-based optoelectrical synaptic memristor that integrates with data storage, optical synapses, logic gates, and neuromorphic computing is demonstrated. The MRS ability of the device is investigated by varying *I*_cc_ and UV light intensities (254 nm). By altering the polarity of the input voltage, the memristor can perform the functions of two logic gates, AND and OR. The device has also demonstrated consistent synaptic characteristics such as PPF, SIDP, SNDP, STDP, and SFDP. When exposed to UV light, the device demonstrated advanced synaptic features such as LTM, STM, and learning-forgetting-relearning. In addition, the fabricated device could achieve a high pattern accuracy (90.7%) in ANN simulations. The integrated functions of optoelectronic memory storage and synaptic learning behavior make it a potential candidate for future in-memory computing systems.

## Materials and methods

### Device fabrication

The Ga_2_O_3_ thin film as an active switching layer was prepared by radio-frequency (RF) sputtering at 300 K. Throughout the sputtering process, the power and the air pressure were 120 W and 0.6 Pa, respectively. The flow rates of nitrogen and oxygen were adjusted to 45 sccm and 5 sccm, respectively. The thickness of the Ga_2_O_3_ thin film utilized in this device is 60 nm. The Ag electrodes are circular with a radius of 50 µm.

### Characterization and measurements

Atomic force microscopy (AFM) was employed for surface morphology analysis. X-ray photoelectron spectroscopy (XPS, PHI VersaProbe 4) was performed to confirm the chemical composition of the Ga_2_O_3_ thin film. The crystalline structures of the Ga_2_O_3_ thin film were analyzed by an X-ray diffractometer (XRD, Bruker D8 Advance). The absorption spectra of the film were measured with a UV–vis spectrophotometer (Lambda950). The electronic and photoelectronic characteristics of the devices were investigated using a Keithley 4200-SCS semiconductor parameter analyzer under both dark conditions and 254/365 nm optical illumination. The ultraviolet light and pulses were generated by an LED controller (HPS-01A, CHIEF ELECTRO-OPTICS Instrument).

### ANN simulation

To investigate the optoelectronic-introduced synaptic behavior, we utilize a basic artificial neural network (ANN) for image recognition. Our work on neuromorphic vision systems is replicated using MATLAB. The training dataset is derived from the Modified National Institute of Standards and Technology (MNIST) database, a substantial collection of handwritten digits frequently employed to train diverse image processing systems. The training details for the ANN are shown in Table S6 (Supplementary Information).

## Supplementary information

Supplementary Information

## Data Availability

The data that support the findings of this study are available from the corresponding author upon reasonable request.
