# Supplementary material for: Versatile optoelectronic memristor based on wide-bandgap Ga2O3 for artificial synapses and neuromorphic computing
Source: Light Sci Appl. 2025 Apr 15;14:161. doi: 10.1038/s41377-025-01773-6 (PMC11997223; doi:10.1038/s41377-025-01773-6)
Supplement: Supplementary file 1 — Supplementary Information [file 41377_2025_1773_MOESM1_ESM.docx]

**Supplementary Information for**

**Versatile optoelectronic memristor based on wide-bandgap Ga_2_O_3_ for artificial synapses and neuromorphic computing**

*Dongsheng Cui,^1,2^ Mengjiao Pei,^3^ Zhenhua Lin,^1,2*^ Hong Zhang,^1,2^ Mengyang Kang,^1,2^ Yifei Wang,^1,2^ Xiangxiang Gao,^1^ Jie Su,^1,2^ Jinshui Miao,^4^ Yun Li,^3*^ Jincheng Zhang,^1,2^ Yue Hao,^2^ Jingjing Chang^1,2^**

^1^Advanced Interdisciplinary Research Center for Flexible Electronics, Academy of Advanced Interdisciplinary Research, Xidian University, 710071, Xi'an, China.

^2^State Key Laboratory of Wide-Bandgap Semiconductor Devices and Integrated Technology, School of Microelectronics, Xidian University, 710071, Xi'an, China.

^3^National Laboratory of Solid-State Microstructures, School of Electronic Science and Engineering, Collaborative Innovation Center of Advanced Microstructures, Nanjing University, 210093, Nanjing, China.

^4^State Key Laboratory of Infrared Physics, Shanghai Institute of Technical Physics, Chinese Academy of Sciences, Shanghai, 200083, China

*Corresponding author Email: jjingchang@xidian.edu.cn; zhlin@xidian.edu.cn; yli@nju.edu.cn

**The power consumption for the multilevel storage and logic gate functions**

**Table S1.** The power consumption of multilevel storage function.

| Muti-level storage | *V*_set_ (v)  (average) | *V*_reset_ (V)  (average) | *P*_set_ (mW)  (average) | | *P*_reset_ (W)  (average) | |
| --- | --- | --- | --- | --- | --- | --- |
| 1mA | 0.87 | -1.42 | 0.87 | 3.38$\times$10^-6^ | |  |
| 0.5mA | 1.16 | -1.21 | 0.58 | 5.56$\times$10^-5^ | |  |
| 0.1mA | 0.94 | -1.37 | 0.09 | 1.67$\times$10^-5^ | |  |
| 0.05mA | 0.72 | -0.81 | 0.04 | 3.20$\times$10^-6^ | |  |
| 0.8 mW/cm^2^ | 0.72 | -0.22 | 0.72 | 2.20$\times$10^-5^ | |  |
| 1.1 mW/cm^2^ | 0.74 | -0.10 | 0.74 | 3.22$\times$10^-9^ | |  |
| 1.4 mW/cm^2^ | 0.40 | -0.08 | 0.40 | 1.81$\times$10^-9^ | |  |
| 1.7 mW/cm^2^ | 0.22 | -0.13 | 0.22 | 4.90$\times$10^-9^ | |  |

The following two equations are used to calculate the power consumption of the multilevel storage function during the set and reset processes.

For the set process: $P_{\mathrm{set}}=I_{\mathrm{set}}\times V_{\mathrm{set}}$

For the reset process: $P_{\mathrm{reset}}=I_{\mathrm{reset}}\times V_{\mathrm{reset}}$

**Table S2.** The power consumption of logic gates.

| Logic Gate | Input | *V*_read_ （V） | *I*_out_ (pA)  (average) | *P* (pW)  (average) |
| --- | --- | --- | --- | --- |
| AND | 00 | -0.1 | 0.23 | 0.023 |
|  | 01 | -0.1 | 19.60 | 1.96 |
|  | 10 | -1 | 5.63 | 5.63 |
|  | 11 | -1 | 53.75 | 53.75 |
| OR | 00 | 0.1 | 0.18 | 0.018 |
|  | 01 | 0.1 | 55.30 | 5.53 |
|  | 10 | 1 | 148.48 | 148.48 |
|  | 11 | 1 | 390.84 | 390.84 |

The formula for calculating power consumption in logic operations is as follows:

$$P_{\mathrm{logic}}=I_{\mathrm{out}}\times V_{\mathrm{read}}$$

**The energy consumption for the optical synapse and neuromorphic computation**

**Table S3.** The energy consumption of the optical synapse.

| Photo-synapse | P_spike_  (mW cm^-2^) | T_duration_  (s) | Energy  (J µm^-2^) |
| --- | --- | --- | --- |
| spike-intensity dependent plasticity (SIDP) | 1.2 | 6 | 7.20$\times$10^-11^ |
|  | 1.5 | 6 | 9.00$\times$10^-11^ |
|  | 1.8 | 6 | 1.08$\times$10^-10^ |
|  | 2.1 | 6 | 1.26$\times$10^-10^ |
|  | 2.4 | 6 | 1.44$\times$10^-10^ |
| spike-number dependent plasticity (SNDP) | 1.2 | 6 | 7.20$\times$10^-11^ |
| spike-time dependent plasticity (STDP) | 1.2 | 1 | 1.20$\times$10^-11^ |
|  | 1.2 | 3 | 3.60$\times$10^-11^ |
|  | 1.2 | 6 | 7.20$\times$10^-11^ |
|  | 1.2 | 9 | 1.08$\times$10^-10^ |
|  | 1.2 | 12 | 1.44$\times$10^-10^ |
|  | 1.2 | 15 | 1.80$\times$10^-10^ |
| spike-frequency dependent plasticity (SFDP) | 1.2 | 25 | 3.00$\times$10^-10^ |

The estimation of energy consumption for the synaptic device is based on the following equations: $E_{\mathrm{programming}}=P_{\mathrm{spike}}\times T_{\mathrm{duration}}$; $E_{\mathrm{erasing}}=V_{\mathrm{read}}\times J_{\mathrm{out}}\times T_{\mathrm{duration}}$,

Here, *E*_programming_ and *E*_erasing_ denote the energy consumption in a single photonic programming process and electric erasing process, respectively. *P*_spike_, and *T*_duration_ are the optical spike power and the spike duration, respectively. *V*_read_ and *J*_out_ represent read voltage and output current density.

**Table S4.** The energy consumption of the optical potentiation in neuromorphic computation.

| Neuromorphic computation | P_spike_  (mW cm^-2^) | T_duration_  (s) | Energy  (J µm^-2^) |
| --- | --- | --- | --- |
| Optical potentiation | 1.8 | 3 | 5.4$\times$10^-11^ |

**Table S5.** The energy consumption of the electrical depression in neuromorphic computation.

| Neuromorphic computation | *V*_read_ (V) | *J*_out_ (nA µm^-2^)  (average) | T_duration_ (s) | Energy (J µm^-2^)  (average) | |
| --- | --- | --- | --- | --- | --- |
| Electrical depression | -1 | 4.84$\times$10^-5^ | 0.1 | | 4.84$\times$10^-15^ |

**ANN Simulation details**

The training details for the ANN are shown below:

**Table S6.** Training details of the ANN.

| **Parameter** | **Values** |
| --- | --- |
| Network architecture | 784-100-10 |
| Number of layers | 3 |
| Training epochs | 200 |
| Activation function | Softmax |
| Learning rate | 0.005 |
| Weights | Device conductance |

**Table S7.** Comparison of recently published Ga_2_O_3_ based optical synaptic devices.

| Materials | Multi-level storage | Logic gate | | Optical synapse | | Neuromorphic computing | | Ref. |
| --- | --- | --- | --- | --- | --- | --- | --- | --- |
| Ga_2_O_3_/MoS_2_ memristor | × | × | √ | | × | | ^1^ | |
| Al_2_O_3_/Ga_2_O_3_ transistor | × | × | √ | | × | | ^2^ | |
| β-Ga_2_O_3_ phototransistor | × | × | √ | | √ | | ^3^ | |
| Ga_2_O_3_ memristor | × | × | √ | | √ | | ^4^ | |
| Ga_2_O_3_:Sn  photosensor | × | × | √ | | √ | | ^5^ | |
| Ga_2_O_3_/MoS_2_ phototransistor | × | √ | √ | | × | | ^6^ | |
| Ga_2_O_3_  /GaN  photosensor | × | √ | √ | | √ | | ^7^ | |
| a-Si:H/a-Ga_2_O_3_ phototransistor | × | × | √ | | √ | | ^8^ | |
| β-Ga_2_O_3_/a-Ga_2_O_3_ photosensor | × | √ | × | | √ | | ^9^ | |
| a-Ga_2_O_3_ memristor | √ | √ | √ | | √ | | This work | |


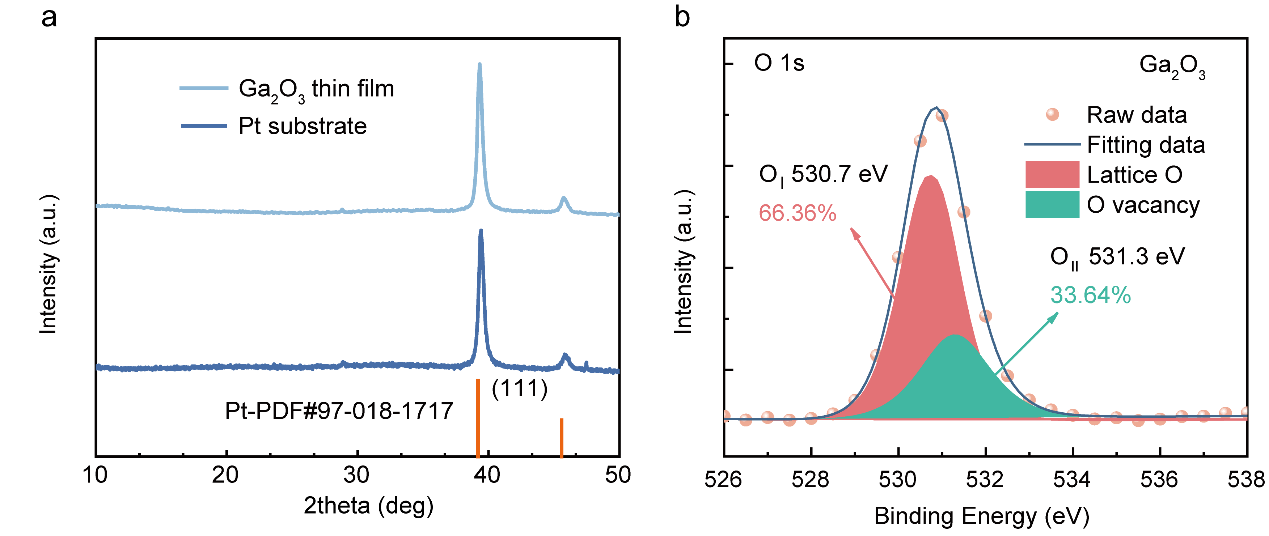


**Fig. S1 Characterization of Ga_2_O_3_ film.** **a** XRD patterns of Ga_2_O_3_ thin film and Pt substrate. **b** XPS O1s spectrum of the Ga_2_O_3_ thin film.


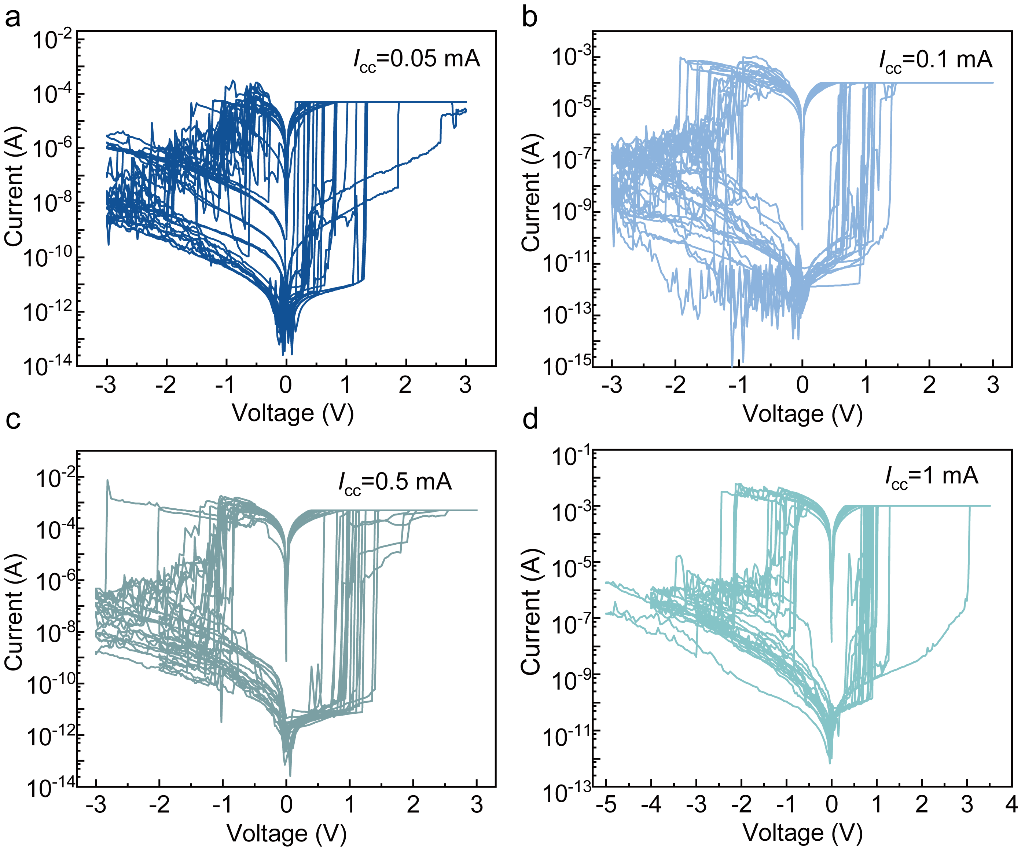


**Fig. S2 *I*-*V* curves of the Ag/Ga_2_O_3_/Pt memory device measured in multiple cycles at different *I*_cc_**. **a** 0.05 mA, **b** 0.1 mA, **c** 0.5 mA, and **d** 1 mA.


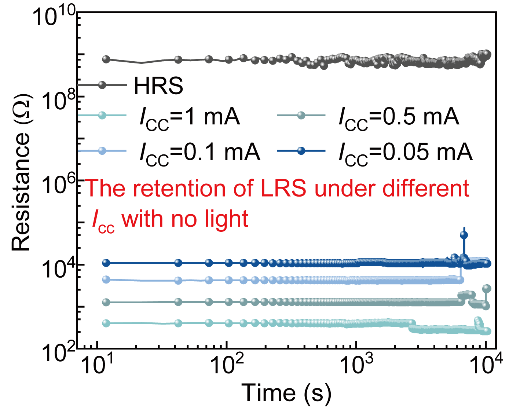


**Fig.S3** Retention characteristics under different *I*_cc_.


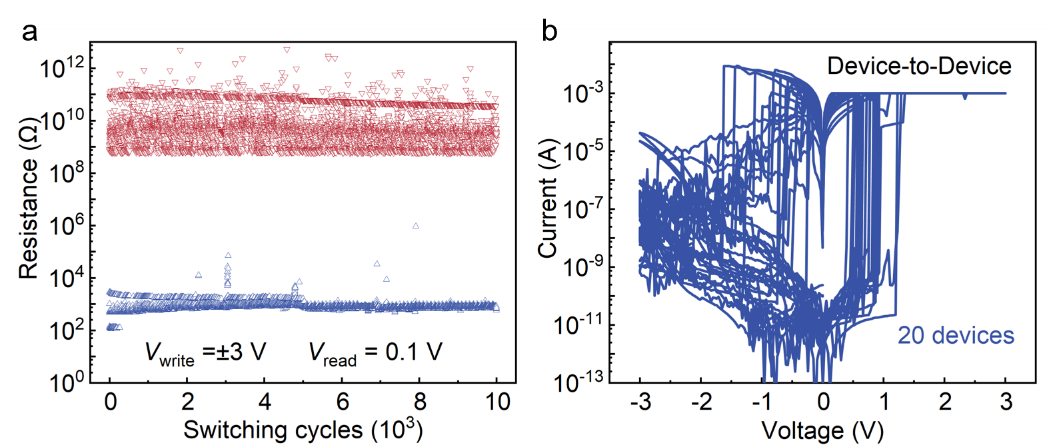


**Fig. S4** The resistive switching characteristics of Ga_2_O_3_-based memristors. **a** The endurance. **b** The device-to-device variability.

**Current Conduction Mechanism and Resistive Switching Mechanism**

**
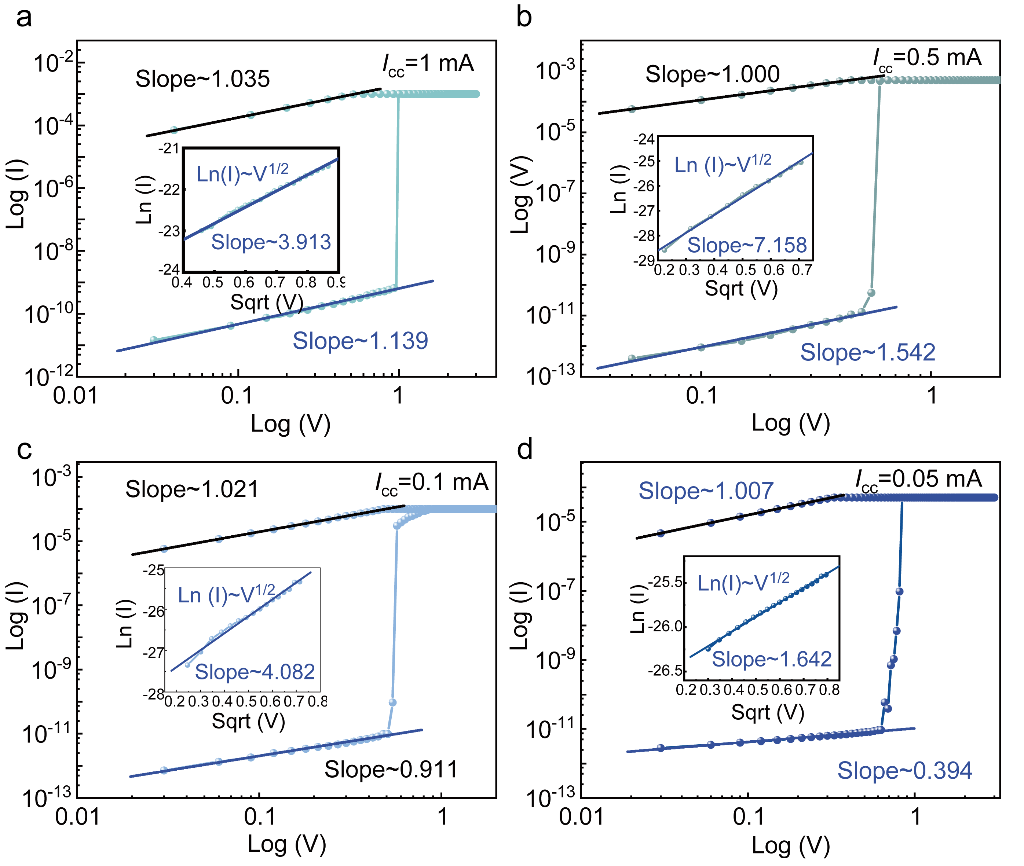
**

**Figs. S5** Log-log curve plots for the Ag/Ga_2_O_3_/Pt device at different *I*_cc_ **a** 1 mA, **b** 0.5 mA, **c** 0.1 mA, and **d** 0.05 mA.

Fig. S5 illustrates the fitting of the double logarithmic curve for varying *I*_cc_ in both the high-resistance state (HRS) and low-resistance state (LRS). In the LRS, the slopes of the fitted curves are 1.035, 1.000, 1.021, and 1.007, respectively, indicating a typical conduction filaments (CFs) mechanism. In the HRS, a linear fit of this data segment is performed based on the ln(*I*) versus *V*^1/2^ relationship. The fitted straight lines have slopes of 3.913, 7.158, 4.082, and 1.642, which are fully consistent with the Schottky emission mechanism.


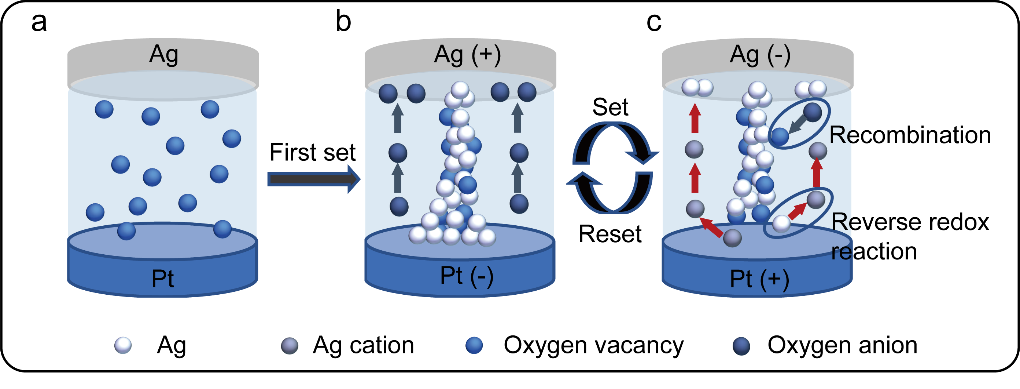


**Fig. S6a-c** The physical mechanism of the resistive transformation process of the Ag/Ga_2_O_3_/Pt device.

The potential RS mechanism: during the set process, a positive voltage is applied to the Ag top electrode (TE), while a negative voltage is applied to the Pt bottom electrode (BE). When the electric field is oriented vertically downward, the Ag electrode undergoes oxidation to produce Ag⁺ ions (Ag → Ag⁺ + e⁻), which migrate from the Ga_2_O_3_ layer to the BE (Pt). Upon reaching the BE, the Ag⁺ ions are reduced to Ag atoms, accumulating to form conical CFs. Simultaneously, the oxygen anions within the Ga_2_O_3_ thin film migrate toward the TE, contributing to the formation of CFs. In the reset process, a forward voltage is applied to the BE, reversing the direction of the electric field upward. At this point, the Ag atoms in the conductive filaments are oxidized at the BE, leading to the rupture of the CFs. Concurrently, the oxygen anions migrate toward the BE to recombine with oxygen vacancies under the electric field, contributing to the rupture of the CFs.


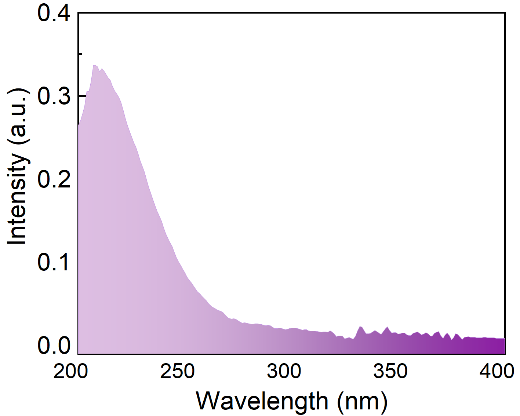


**Fig. S7** The absorbance of a-Ga_2_O_3_ thin film for different wavelengths of light.


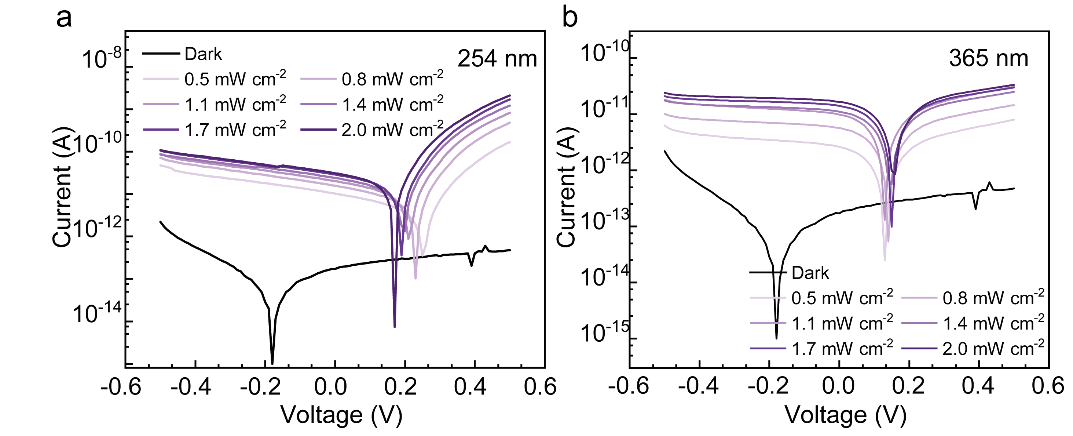


**Fig. S8 Optical response of the device under different UV light.** **a** 254 nm and **b** 365 nm.


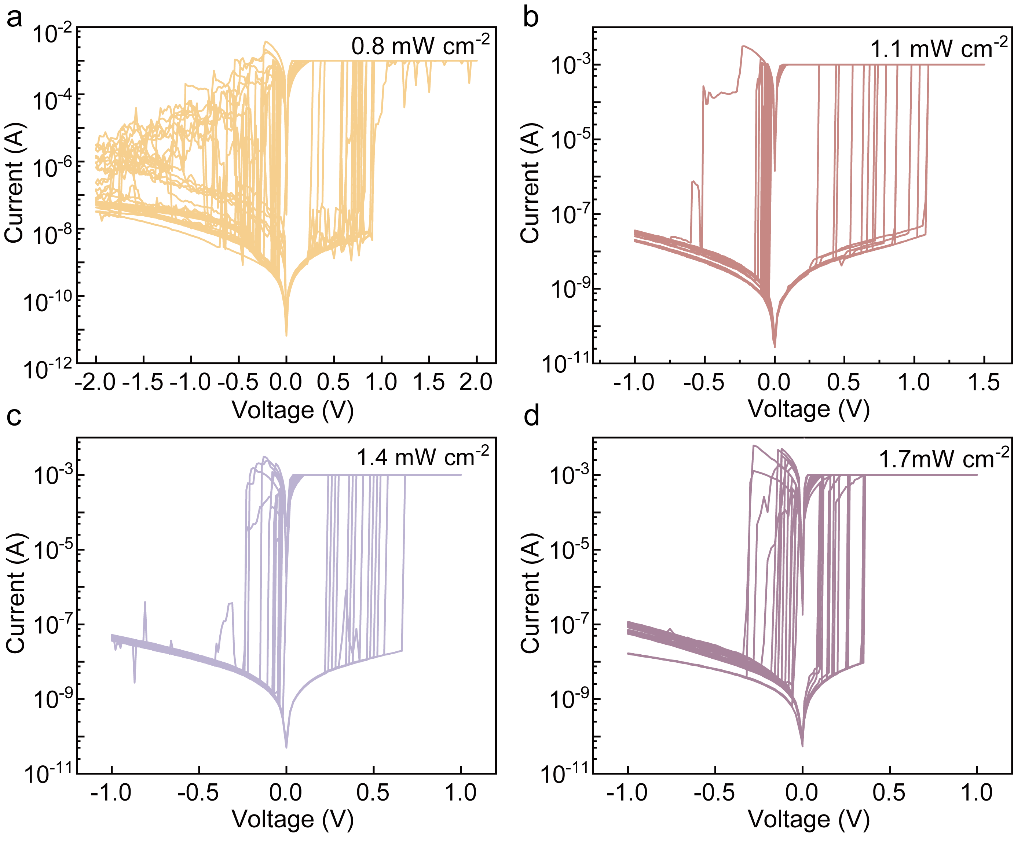


**Fig. S9** ***I*-*V* curves of the Ag/Ga_2_O_3_/Pt memory device measured under different light intensities (254 nm)**. **a** 0.8 mW cm^-2^, **b** 1.1 mW cm^-2^, **c** 1.4 mW cm^-2^, and **d** 1.7 mW cm^-2^.


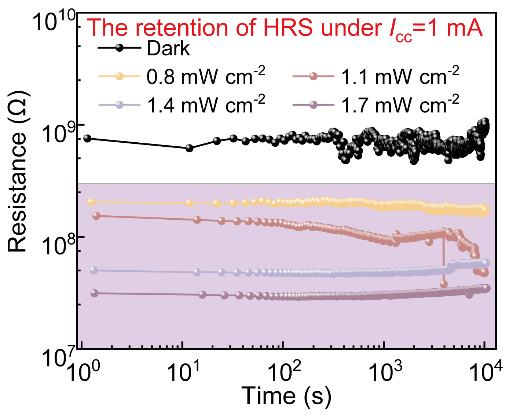


**Fig. S10** Retention characteristics under different UV intensities.


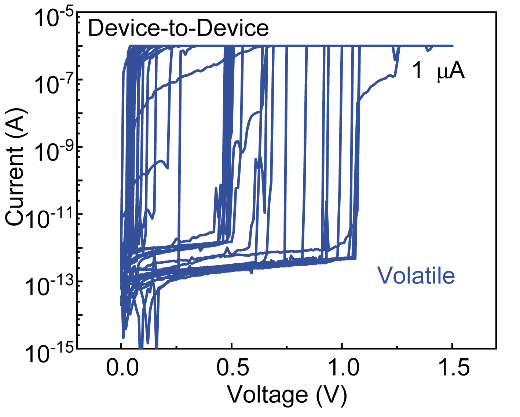


**Fig. S11** *I*-*V* curves of 20 devices with *I*_cc_=1×10^-6^ A.


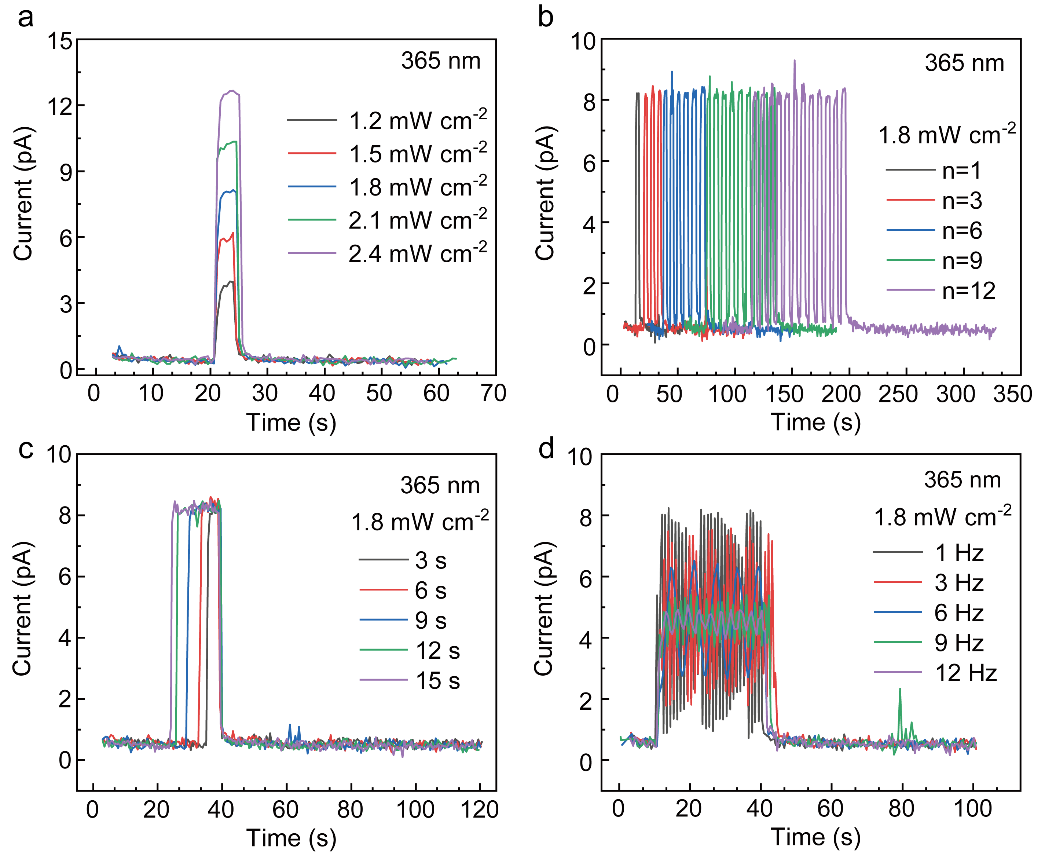


**Fig. S12 Photo-responsive currents measured under different conditions**. **a** intensity, **b** number of light pulses, **c** duration, and **d** frequency. (UV light: 365 nm)


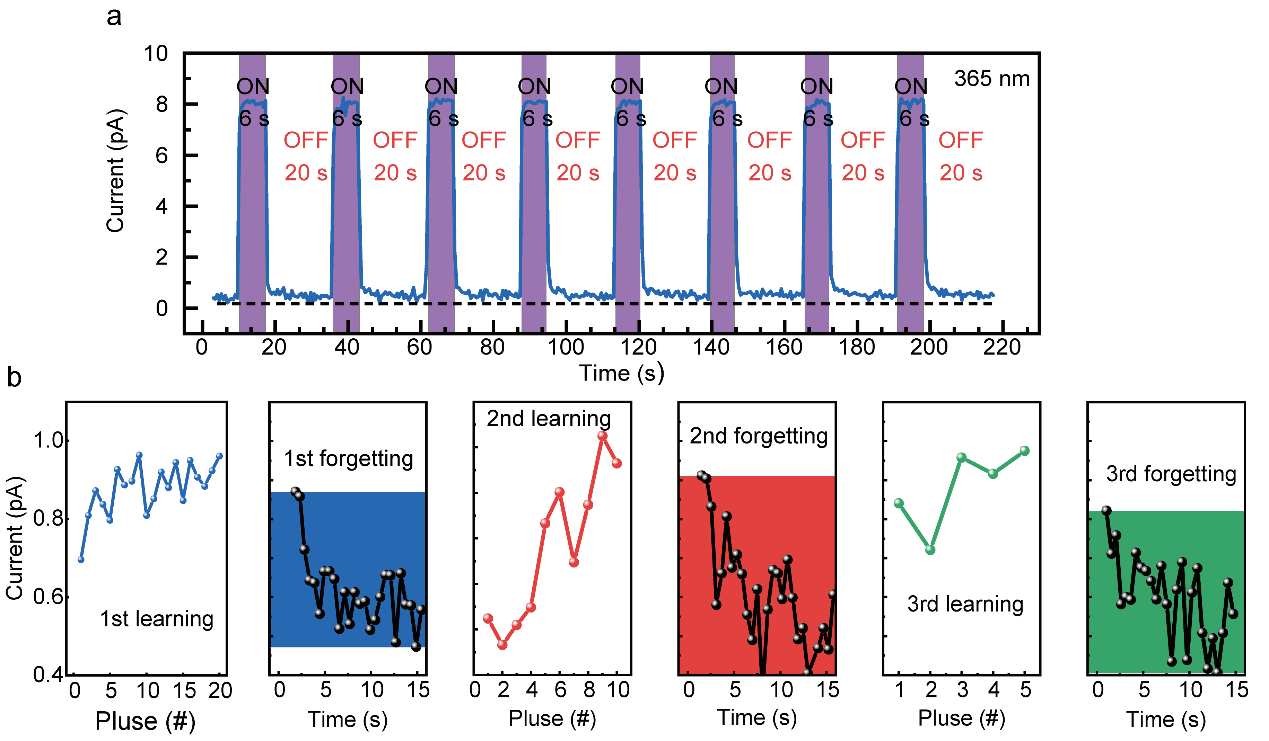


**Fig. S13 Learning-forgetting-relearning behavior under 365 nm.** **a** Learning-forgetting-relearning behavior with seven cycles (365 nm). **b** The "learning experience" behavior was measured under 365 nm light pulse stimulation. Light intensity: 1.8 mW cm^-2^, pulse width: 3 s, pulse interval: 3 s.

Reference:

1. Li, R. *et al.* Multi-modulated optoelectronic memristor based on Ga_2_O_3_/MoS_2_ heterojunction for bionic synapses and artificial visual system. *Nano Energy* **111**, 108398 (2023).

2. Jo, E. S. & Rim, Y. S. Assessment of trapping layer control in IGZO/Al_2_O_3_/Ga_2_O_3_ synaptic transistor for neuromorphic computing. *Materials Today Physics* **37**, 101194 (2023).

3. Yoon, Y., Kim, Y., Hwang, W. S. & Shin, M. Biological UV Photoreceptors-Inspired Sn-Doped Polycrystalline *β*-Ga_2_O_3_ Optoelectronic Synaptic Phototransistor for Neuromorphic Computing. *Adv Electron Mater* **9**, 2300098 (2023).

4. Kim, J. H. *et al.* Oxide Semiconductor Memristor-Based Optoelectronic Synaptic Devices With Quaternary Memory Storage. *Adv Electron Mater* **10**, 2300863 (2024).

5. Li, P. *et al.* Tin Doping Induced High-Performance Solution-Processed Ga_2_O_3_ Photosensor toward Neuromorphic Visual System. *Adv Funct Mater* **33**, 2303584 (2023).

6. Zhang, Y. *et al.* Optoelectronic Neuromorphic Logic Memory Device Based on Ga_2_O_3_/MoS_2_ Van der Waals Heterostructure with High Rectification and On/Off Ratios. *Adv Funct Mater* (2024) doi:10.1002/adfm.202408978.

7. Feng, S. *et al.* Dual-Mode Conversion of Photodetector and Neuromorphic Vision Sensor via Bias Voltage Regulation on a Single Device. *Advanced Materials* **35**, 2308090 (2023).

8. Yoon, Y., Kim, Y., Choi, S., Kwon, J. & Shin, M. Synaptic a-Si:H/a-Ga_2_O_3_ phototransistor inspired by the phototaxis behavior of organisms with all-optical and all-electrical stimulation modes. *Nano Res* **17**, 7631–7642 (2024).

9. Ye, J. *et al.* Photocurrent Ambipolar Behavior in Phase Junction of a Ga_2_O_3_ Porous Nanostructure for Solar-Blind Light Control Logic Devices. *ACS Appl Mater Interfaces* **16**, 26512–26520 (2024).

10. Kang, J. *et al.* Cluster-type analogue memristor by engineering redox dynamics for high-performance neuromorphic computing. *Nat Commun* **13**, 4040 (2022).

11. Khot, A. C. *et al.* Amorphous Boron Nitride Memristive Device for High-Density Memory and Neuromorphic Computing Applications. *ACS Appl Mater Interfaces* **14**, 10546–10557 (2022).

12. Cui, D. *et al.* Coexistence of Bipolar and Unipolar Resistive Switching Behavior in Amorphous Ga_2_O_3_ Based Resistive Random Access Memory Device. *IEEE Electron Device Letters* **44**, 237–240 (2023).
